# Ability or luck: A systematic review of interpersonal attributions of success

**DOI:** 10.3389/fpsyg.2022.1035012

**Published:** 2023-01-05

**Authors:** Odessa S. Hamilton, Grace Lordan

**Affiliations:** The Inclusion Initiative, Department of Psychological and Behavioural Science, London School of Economics and Political Science, London, United Kingdom

**Keywords:** luck, ability, interpersonal, attributions, success, gender, race

## Abstract

The role of luck in success has a relatively minor, albeit consistent history in academic discourse, with a striking lack of literature engaging with notions of luck within occupational environments. Elucidating why people attribute their own success to luck over ability has predominated in the literature, with interpersonal attributions receiving less attention. Here, we center on systematically summarizing the evidence on interpersonal attributions of success as a function of ability or luck, with a particular focus on whether these differs by gender and race. The perception of the success of others from different sociodemographic groups, and how it is attributed, is a crucial leverage point for inclusion and diversity. Particularly as women and ethnic groups continue to be systematically disadvantaged in the workforce. Ignoring the role of luck conceals and augments *privilege*, even if not deliberately or consciously invoked. Using the Prisma protocol, this review offers evidence from experiments, published between 1970 and 2020, derived from five electronic bibliographic databases; Business Source Complete; PsychINFO; Scopus; Web of Science; and Google Scholar. There were a limited number of studies on gender that found an effect, but with few exceptions, the papers that pertain to race converged on the understanding that interpersonal attributions of success were predicated on this immutable factor. Such that black individuals were more often viewed as lucky in their success and less able, which translates to lesser opportunity and reward. Decades of research have pointed to individuals making systematic attribution errors in success by gender and race; this review only partially substantiates this consensus and provides narrow support for the view that those believed to be the most talented in society may merely be the luckiest. We add to evidence that context matters.

## Introduction

Individuals engage in sensemaking to navigate a complex and dynamic world. As part of this process individuals construct conceptual attributions to simplify, predict, control and master phenomena they encounter (Heider, 1958). Information about temporal consistency, distinctiveness and consensus across individuals are systematically processed to arrive at a causal attribution for an observed effect (Regan and Totten, 1975). Thus, attributions offer a causal explanation for, and interpretations of, experienced or observed events (Christophe, 2016), such as success, which is a widely rationalized phenomenon (Frank, 2016). The notion of luck and the idiosyncratic nature of how individuals attribute the cause of success to luck over ability is focal to attribution research (e.g., Luginbuhl et al., 1975; Pritchard and Smith, 2004; Liu, 2021).

The perception and subsequent ascription of success has important implications for occupational engagement. Individuals frequently conflate luck with ability evaluations and ignore how future performance tends to regress to the mean (viz., veering toward a statistical average), so misattributions of luck in performance can develop into false expectations on either side of the performance spectrum (Denrell et al., 2019). Unfortunately, a satisfactory counterfactual estimation cannot be performed to assess whether the same success would have occurred under different conditions (Osterloh and Frey, 2019). These misattributions, if they systematically differ by sociodemographic characteristics, matter for labor market outcomes, such as recruitment decisions, pay, promotions and opportunities (Liu, 2021). Therefore, we contribute novel evidence to the literature by systematically reviewing a combination of experiments, conducted in the laboratory and in the occupational field, that consider whether attributions of success by luck and ability vary by gender and race in a variety of contexts.

The importance of our study is bellied in the tendency for the narrativization of chance and the consolidation of luck being key explanatory factors in making sense of success (Loveday, 2018). These biases also occur at the organizational level, but the potential of attribution theory to contribute to organizational sciences has not been realized (Martinko and Mackey, 2019). Organizations afford most opportunity to those who are successful. At face value this seems justified, but they may not be drawing reliable conclusions about what leads to such success (Balasubramanian et al., 2022). The role of luck when advancing in one’s career is captured by Berger et al.’s (1966) Expectation States Theory, which highlights that opportunity for success is conferred to individuals on a spurious expectation of presumed future successes, often predicated on little more than an assumption of immutable dispositional, yet diffuse characteristics. This leaves the door open for misattributions of ability that differ by sociodemographics and distort a fair advancement process.

Attribution processes have also emerged as an important consideration in how leaders interact with their employees (Martinko and Mackey, 2019). The emotional disposition of the *observer* and empathy toward the *actor* is said to be critical to the direction of the attribution. Causal attributions are more situational/luck orientated and less dispositional/ability oriented when *observers* took the perspective of *actors*. This suggests a likelihood that attributions will favor people who are sociodemographically similar to the observer (Kelley, 1967).

According to Weiner’s (1979) Causal Attribution Model, the most salient determinants of success are ability, effort, task-difficulty, and luck, which are treated quantitatively. These can be analyzed along three dimensions of stability – the temporal nature of a cause; controllability, or the degree of volitional influence one has over a cause; and perceived *locus of control* – attributing events to oneself (i.e., internally) or the environment (i.e., externally; Rotter, 1966; Spector, 1982). Using this framework, the factor of luck in success is perceived as unstable, uncontrollable and external (Yan and Gaier, 1994; Brun et al., 2021). However, this method has received criticism as the dimensions are antithetical to the way we truly think about life outcomes, and in reality, qualitative assessments drive complex responses about why one believes another has enjoyed success or experienced failure (Hill and Augoustinos, 1997). Still, Weiner’s model offers a common-sense framework through which we quantitatively measure and report inter-individual differences in attributions. Because Weiner’s model has been widely influential, it also serves as a useful tool to make comparisons across studies that otherwise have vastly different designs.

One sociodemographic factor of particular interest to the study of interpersonal success attributions is gender. Gender is not only a highly salient sociodemographic, but there are several further reasons that suggest that success attributions, particularly in professional contexts, might be gender sensitive. Gender-role stereotypes are the rudiments of gender discrimination narratives, and are especially tenacious, with a powerful tendency to maintain them (Heilman and Haynes, 2005). Historically, men have been thought to be more competent than women, which is a clear disadvantage to women seeking managerial, executive, or male-dominated roles, despite information contradictory to stereotypes (Heilman and Guzzo, 1978). Psychologists and feminists alike have argued that the concept of success is epitomized through maleness (Jabes, 1980). Therefore, if a woman is successful, this unexpected outcome is explained by luck (Deaux and Emswiller, 1974) or other mutable, unstable causes (Feldman-Summers and Kiesler, 1974). There is substantive evidence to support that these stereotypes persist in occupational settings (e.g., Dodge et al., 1995; Powell, 1999; Schein, 2001).

Hill and Augoustinos (1997) describe gender-based attribution differentials as a ‘*male-favoring*,’ ‘*female-derogating*’ phenomena, where males are favored under circumstances that are both stereotypically masculine and feminine orientated or in neutral conditions. The suggestion is that attributions of success can be skewed by an awareness of an individual’s gender, such that a man’s success is normatively attributed to ability without regard to indices of luck, whereas the success of a woman is normatively derogated through dominant attributions of luck (e.g., Deaux and Emswiller, 1974; Etaugh and Brown, 1975; Feather, 1975). This can be regarded as an instance of the “*ultimate attribution error*” (Pettigrew, 1979) with respect to gender. This systematic attribution error provides the basis for gender discrimination reinforced by luck attributions within occupational settings, and the ramifications of these inferences are multidimensional. Males can become disproportionately rewarded for what is thought to be ability, without regard to the ease through which earlier successes may have been afforded. This can also be harmful to men when hired or promoted into roles where failure is foreseeable (Hohman and Brown, 2020). In contrast, women may be overlooked for employment or promotion because of a perceived rather than actual ability-deficit. This error in judgment can cause women to be deemed unworthy of the positions they hold, excluded from key discourse, given less credit, and overlooked for progression into senior roles (Heilman and Haynes, 2005; Heilman, 2012). It can also mean that individual successes in women are thought of as outliers that cannot be generalized to women more broadly. Such pretenses can influence quantifiable metrics, with women less likely than men to be rewarded with equitable pay, merited salary increases, or promotions (e.g., Heilman and Guzzo, 1978; Blackaby et al., 2005; Auspurg et al., 2017).

Heterogeneity in attributions between the sexes can drastically shape intrinsic and occupational gendered experiences; with males seen as being instrumental in their success and women seen as being passive (Sieverding and Koch, 2009). Finally, known unmerited reward systems in male-dominated fields can discourage female participation for fear of their progression being hindered for reasons other than a lack of ability. These collectively reduce the chances of equitable representation into roles that have been historically reserved for men (Powell, 1999). For these reasons, the role of gender in success attributions in professional contexts is a focal point of our review.

Another highly salient sociodemographic widely hypothesized to be central to interpersonal success attributions in professional settings is race. Ethnic groups, particularly black members, are systematically disadvantaged in the workforce, and remain notably underrepresented in corporate environments in senior positions. Organizations tend to be, instead, distributed in such a way that these groups are predominately relegated to subordinate positions, with little prospect of upward mobility (Orpen, 1981; Knight et al., 2003). The proliferation of workforce inclusion initiatives has made attributions of success potentially more vulnerable to implicit and explicit biases because the working environment has become more diverse. These biases can negatively impact chances of employment and opportunities for advancement, with the potential inadvertent diminishing of performance and future success (Knight et al., 2003; Ellis et al., 2006).

Ingroup-outgroup biases are such that members tend to view others within their group in favorable terms, but then look unfavorably toward individuals outside of their group (Whitehead et al., 1982). Race is one distinguishing feature that is particularly conducive to ingroup-outgroup bias. This bias may be a result of negative disposition toward others unlike us, or stereotypes that have integrated into our belief system. Again, Pettigrew’s (1979) theory on the “*ultimate attribution error*” elucidates this propensity for misattributions toward outgroup members well. Such that individuals judge the success of those who differ from them more harshly, attributing their success to factors beyond their control, such as luck. It explains why under these circumstances ethnic groups are seen as less competent, with less potential. For these reasons, ethnicity is included in this review as a complement to gender. We explore a number of studies that investigate whether a homogeneity exists between attribution outcomes made by different racial groups. In particular, we focus on situational attributions (i.e., luck) and dispositional attributions (i.e., ability) made for white individuals as compared to other ethnic individuals.

Overall, this study aims to summarize the current understanding of the conditions in which individuals in society attribute the successes of others to luck, and how underlying biases in attributions can result in disparities between opportunities afforded or denied to people dependent on their gender or ethnicity. One meta-analysis by Swim and Sanna (1996), with data between 1973 and 1993, builds on earlier qualitative reviews that have examined gender differences in attributions. But their study yielded mixed results and small effects sizes that the authors conclude are plausibly a result of measurement artifact. More recently, Brun et al.’s (2021) meta-analytic study, involving 15,213 participants across 43 studies, found that Weiner’s (1979) causal dimensions influenced behaviors and performance through psychological consequences. They concluded that event valence is central to attribution evaluations. However, their approach centers on the intrapersonal level of Weiner’s (1979) attributional theory, focusing on how individuals explain their own success or failure. Thus, the area of research that focuses on interpersonal attributions warrants further enquiry, particularly given the notable shift in prejudices and societal expectations over time (Charlesworth and Banaji, 2022). Furthermore, our review is the first of its kind to summarize extant literature focused on interpersonal attributions, with a view to elucidate whether attributions of success are differentially ascribed to luck or ability, as a function of sociodemographic factors that are highly salient, but extraneous to actual performance; namely gender and race.

## Methods

### Systematic review strategy

We followed the review protocol Prisma, which can be accessed at www.prisma.com. A comprehensive search for all available research was performed in five electronic bibliographic databases. In attempt to counteract the American-European bias, Business Source Complete, PsychINFO, Scopus, Web of Science and Google Scholar were screened. Only peer-reviewed published or *in press* papers, written in English, across the last 50 years (1970–2020) were examined.

**FIGURE 1:**
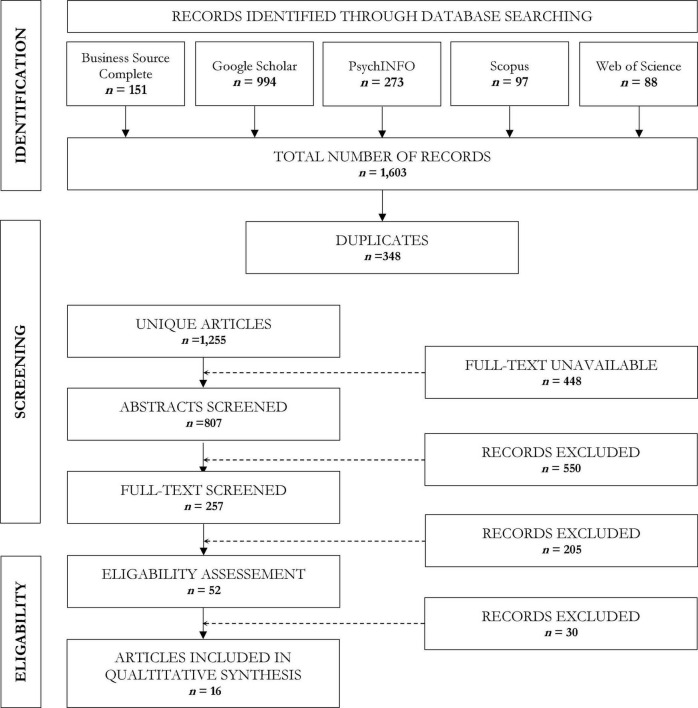
Flow diagram.

There were two strands to the digital search strategy (Effective date: October 20, 2020) to ensure comprehensive data location. The first was to apply search terms to the bibliographic databases. The second strand was to reference earlier review articles published in this field. The papers that were identified from the initial electronic search process were imported into Zotero (version 5.0.96.2). Duplicates were removed. Only primary publications were included. Where relevant, subsequent publications were scrutinized to address any ancillary issues. Criteria for inclusion was as follows: (1) English language, full-length empirical publication, in a peer-reviewed journal; (2) cross-sectional or prospective evidence; (3) sample derived from an educational setting (i.e., laboratory-based) or from an occupational environment (i.e., field-based); (4) participants must be aged 16 years or older; (5) singular focus on interpersonal attribution, or dual focus on interpersonal and intrapersonal attributions. Criteria for data exclusion was as follows: (1) literature was a review, meta-analysis, author manuscript or letter to an editor; (2) results comprised contemporaneous correlations alone; (3) intrapersonal attribution was the sole focus; (4) no reference to indices of luck were made in the results. Criteria for in title or abstract search terms included: (1) “Success*”; “*achievement*”; “*performance*” (2) “Luck*”; “*serendipity*”; “fortune*”; “*chance*”; (3) “*Attribution*”; “*ascription*”; “*cause*”; (4) “*Interpersonal*”; “*observer*”; “*social*”; “*evaluation.*” Domain terms were combined with the “*OR*” Boolean operator. Between domains, terms were combined with the “*AND*” Boolean operator. Titles were screened to determine if they met the inclusion and exclusion criteria. Where titles were insufficient for this purpose, abstracts were screened. Full-text screening was performed on potentially relevant studies that were identified as meeting the criteria, or for which criteria could not be established (Figure 1). Resulting studies were demarcated by their relevance into three sociodemographic categories (i.e., gender; race; and intersectionality) and addressed by ascending year of publication. A reliability study was performed by an independent assessor, who reviewed a randomly selected subset (15%) of the unique articles retrieved in the database search. The selections made by the assessor were compared to the initial determinations and disputes were settled by consensus. 16 papers were derived after full screening and eligibility checks. Data was extracted from each report for in-text summary and subsequent discussion, in addition to a tabulated synopsis (Table 1).

**TABLE 1:** Summary of review articles with sample characteristics (*N* = 4,182).

Category	References	Title	Study count	Methodology	Sample	Participant characteristics	Attribution method	Luck	Ability	Results summary
Gender	Feldman-Summers and Kiesler, 1974	Those who are number two try harder: the effect of sex on attributions of causality.	2	ANOVA 2 × 2 × 3 factorial design	114	50:50 male/female university students	Explicit/direct	×	×	There was no impact of luck as a function of gender. *Observers* expected males to perform better than females on an intellectual task. Females were attributed greater effort than males, but ability, task-difficulty and luck did not interact with *actor* success and gender.
				ANOVA 2 × 2 × 2 × 2 factorial design	160	50:50 male/female University Students	Explicit/direct	×	✓	There was no impact of luck as a function of gender. Male *observers* believed female physicians were less able and had an easier task than male surgeons, but female *observers* believed female physicians had a harder task.
Gender	Heilman and Guzzo, 1978	The perceived cause of work success as a mediator of sex discrimination in organizations.	1	ANOVA 2 × 4 factorial design	29	15:14 male/female masters’ students	Explicit/direct	×	×	There was no impact of luck as a function of gender. Irrespective of the *actors’* gender, dimensions of luck, effort and task-difficulty that are typically ascribed to females, received less reward than attributions of ability that end to be ascribed to males.
Gender	Sherrod and Goodman, 1978	Effects of sex-of-observer on female actors’ causal attributions for success and failure.	1	ANOVA 2 × 2 × 2 factorial design	120	24:96 male/female college students	Implicit/indirect	×	✓	There was no impact of luck as a function of gender. Male *actors* received greater dispositional attributions than female *actors*.
Race	Greenberg and Rosenfield, 1979	Whites’ ethnocentrism and their attributions for the behavior of blacks: A motivational bias.	1	MANOVA 1 between; 2 within	116	White males university students	Explicit/direct	×	✓	There was an impact of luck as a function of race. The more ethnocentric *observers* were, the more they credited white *actors* more for their own success.
Gender	Jabes, 1980	Causal attributions and sex-role stereotypes in the perceptions of women manager.	1	ANOVA 2 × 2 × 3 factorial design	114	Female managers private/public sector (*M*_age_ = 35; 22–61)	Explicit/direct	×	✓	There was an impact of luck as a function of gender. There were only gender main effects. Female *actors* were considered more successful than males. Male *actors* were thought to be luckier and to have had easier tasks than females.
Race	Orpen, 1981	Causal attributions for the success and failure of black and white manager.	1	ANOVA	136	White, male managers, commercial firm (*M*_age_ = 32.6)	Explicit/direct	✓	×	There was an impact of luck as a function of race. The success of white *actors* was predominately attributed to ability and effort, but the success of black *actors* was attributed to luck and the ease of the job.
Race	Whitehead et al., 1982	The effect of subject’s race and other’s race on judgments of causality for success and failure.	1	ANOVA 2 × 2 × 2 factorial design	364	156:208 black/white junior and senior high school students	Explicit/direct	✓	✓	There was an impact of luck as a function of race. An ingroup bias caused a dislike of the outgroup in the failure condition, but this did not translate to the success condition. Black *observers* attributed more ability to black *actors* when success was academic, but white attributions were not influenced by race.
Intersectionality: *gender and race*	Yarkin et al., 1982	Blacks and women must try harder: stimulus persons’ race and sex attributions of causality.	1	ANOVA 2 × 2 × 2 between-subjects factorial design	120	60:60 male/female university students	Explicit/direct	✓	✓	There was no impact of luck as a function of gender. Male and female *observers* attributed greater ability, less effort, and less luck to white males, than white female *actors* or black male and female *actors*.
Gender	Reid et al., 1986	Attribution and sex differences in the employment interview.	1	ANOVA 2 × 2 × 3 factorial design and stepwise regression	180	89:91 male/female university students	Explicit/direct	✓	×	There was an impact of luck as a function of gender. Male *observers* attributed the success of males and female *actors* differentially, as a function of job characteristics. Female *observers* made no such distinction. Former attributions of ability and task-difficulty influenced subsequent attributions.
Gender	Almeida and Kanekar, 1989	Causal attributions for success and failure as a function of sex and job status in India.	1	ANOVA 2 × 2 × 2 × 2 factorial design	336	Male and female university students	Explicit/direct	✓	×	There was an impact of luck as a function of gender. The success of male *actors* in high-status roles was attributed to ability and effort, as compared to females. Female success was only attributed to ability in low-status roles.
Intersectionality: *gender and race*	Greenhaus and Parasuraman, 1993	Job performance attributions and career advancement prospects: an examination of gender and race effects.	1	ANOVA 2 × 2 × 2 × 2 factorial design and multiple regression	1,628	814:814 black/white supervisors	Explicit/direct	×	✓	There was a conditional impact of luck as a function of gender, and there was an impact of luck as a function of race. Among highly successful managers, females were less likely to receive ability attributions than males. Black managers were less likely to received attributions of ability and effort, with a greater salience toward situational attributions than white managers. Race differences attenuated as interpersonal familiarity increased.
Gender	Taylor et al., 1993	Gender and attribution: a reversal of bias?	1	ANOVA 2 × 2 × 2 factorial design	120	60:60 males/fmales (aged = 14–50) black/white/hispanic Professionals	Explicit/direct	×	×	There was no impact of luck as a function of gender. Success was predominately attributed to ability, irrespective of the *actors’* gender. No other interactions or main effects were demonstrated in the success condition. The results suggest that male type-casted roles required more ability than female type-casted roles.
Gender	Hill and Augoustinos, 1997	Re-examining gender bias in achievement attributions.	1	ANOVA 2 × 2 × 3 × 2 × 4 factorial design	106	28:78 male/female university students (*M*_age_ = 21.59 ± 5.12)	Explicit/direct	×	✓	There was no impact of luck as a function of gender. There were no differences in attributions made for the success of males and females, but gender-casted courses were perceived differentially. Feminine courses were attributed to less ability and easier task than masculine and neutral courses.
Race	Knight et al., 2003	Out of role? out of luck: the influence of race and leadership status on performance appraisals	1	MANOVA 2 × 2 × 2 factorial design	156	White university students	Implicit/indirect	✓	✓	There was an impact of luck attributions as a function of race. Congruent with race stereotypes, *observers* evaluated black leaders and white subordinates negatively, but white leaders and black subordinates positively. *Observers* used innocuous mistakes of the past to justify negative evaluations of black leaders.
Race	Ellis et al., 2006	The effects of team leader race on performance evaluations: an attributional perspective	1	Regression ANOVA	177	54:123 male/female 15:162 black/white university students	Explicit/direct	×	×	There was a reversed impact of luck attributions as a function of race. Team leader performance and race was a major determinant of subordinate success attributions. In high performing teams, attributions toward black leaders were ability and effort. In low performing teams, attributions toward black leaders were luck and ease of task. Leaders received higher ratings when success was attributed to dispositional characteristics.
Gender	Sieverding and Koch, 2009	Self-evaluation of computer competence: how gender matters	1	Chi-square analysis, ANOVA 2 × 2 and MANOVA 2 × 2	206	99:107 male/female University students (*M*_age_ = 23.7 ± 4.2)	Explicit/direct	×	×	There were no systematic gender differences between attributions of success.

ANOVA, analysis of variance; MANOVA, multivariate analysis of variance; *M*_age_, mean age; ±, standard deviation; ✓, effect observed; ×, effect not observed.

## Results

Most studies leveraged attributions explicitly on self-defined characterizations of ability, effort, task-difficulty, and luck. But there were some instances where luck was implicitly evoked; Knight et al. (2003) for example, proxied luck by stereotype-consistent and stereotype-contrastive factors beyond the *actors’* control. Explicit (i.e., direct) verses implicit (i.e., indirect) inferences of luck have been indicated in Table 1.

Overall, we find that *observers* tend to ignore the existence of situational factors (i.e., luck), with the belief that they are weighting exclusively on personal qualities (i.e., ability) (Table 2). This reliance on dispositional attributions for what may be largely situational effects creates a fallacy, given that success is fundamentally a weighted byproduct of both dispositional and situational factors. The review also indicates that inter-individual demographics directly influence how attributions are made, with a tendency to disadvantage women and Black individuals. Thus, our findings suggest that differential attributions of luck may contribute to differences in high stakes outcomes such as pay, promotions and opportunity gaps. We argue that the perception of success and how it is attributed is a crucial inclusion and diversity leverage point.

**TABLE 2:** Summary of review articles.

Design feature	Papers *N* (%)
**Sociodemographic category**	
Gender	9 (56.25)
Race	5 (31.25)
Intersectionality – *Gender and race*	2 (12.50)
**Sample geography**	
Africa	1 (6.25)
America	12 (75.00)
Asia	1 (6.25)
Australia	1 (6.25)
Europe	1 (6.25)
**Journal**	
*Australian Journal of Psychology*	1 (6.25)
*Canadian Journal of Behavioral Science*	1 (6.25)
*Computers and Education*	1 (6.25)
*Journal of Occupational Behavior*	1 (6.25)
*Journal of Personality*	2 (12.50)
*Journal of Personality and Social Psychology*	1 (6.25)
*The Journal of Social Psychology*	3 (18.75)
*Organizational Behavior and Human Decision Processes*	1 (6.25)
*Organizational Behavior and Human Performance*	1 (6.25)
*Personality and Social Psychology Bulletin*	2 (12.50)
*Sage Journals*	1 (6.25)
*The Irish Journal of Psychiatry*	1 (6.25)

## Gender

Twelve studies among 11 papers investigated gender as an influencer of interpersonal attributions of success, with two papers sitting on the intersect with race. Six of those studies were randomized control studies (RCT) and one was a matched case control study. Nine studies were conducted in the laboratory and three were occupational field-based. A result summary can be found in Table 1. A discussion ordered by the date of publication follows. Our focus is on situational attributions (i.e., luck) and dispositional attributions (i.e., ability) of success made for females as compared to males.

The first paper received on this topic was by Feldman-Summers and Kiesler (1974) who found no difference in attributions to luck as a function of gender in two laboratory studies that investigated the effect of gender on interpersonal attributions. Their study involved 114 participants, who self-identified as male and female, with 10 logical and mathematical exercises, and six answer sheets reflecting fictitious *actor* responses. Participants were advised they were to observe two highly successful, two moderately successful, and two unsuccessful *actors*. At each level of success, *actors* identified as either male or female by way of sex-casted names. Attributions were explicitly made on self-defined characterizations of ability, effort, task-difficulty, and luck. Participants were advised that their evaluations would determine the future involvement of each *actor*. A 2 (*observer*_sex) × 2 (*actor*_sex) × 3 (*actor*_success) analysis of variance (ANOVA) revealed highly significant effects for the level of success (*p* < 0.001). Perceived success increased as the number of problems increased [*F*(2,224) = 1169.88, *p* < 0.010]. As the level of success increased, so did attributions of ability (*F* = 728.64), effort (*F* = 463.77), task-difficulty (*F* = 463.77), and luck (*F* = 30.55). A significant main effect was found for the sex of an *actor* on effort [*F*(1,112) = 4.75, *p* < 0.050], but not for ability, task-difficulty, or luck.

Feldman-Summers and Kiesler’s (1974) second laboratory-based study provided 160 male and female participants with descriptions of highly successful pediatrician or surgeon *actors*. *Actors* identified as either male or female using sex-casted names. The pre-test data suggested that males were not expected to succeed as pediatricians over women but were expected to succeed more as surgeons. Attributions were explicitly made on self-defined characterizations of ability, effort, task-difficulty, and luck, by the division of a circle representing the percentage weight of the *actors’* success. But to invoke the luck condition, half of the descriptions stated that the *actor* secured their practice through nepotism. There was a significant interaction between *actor* sex and the luck condition [*F*(1,155) = 5.572, *p* < 0.050], such that males altered their attributions toward luck more than females did when the luck condition was invoked. The 2 (*observer*_sex) × 2 (*actor*_sex) × 2 (*actor*_specialty) × 2 (acceded practice) ANOVA revealed both sexes were expected to be equally successful as pediatricians. However, males were expected to be more successful as surgeons than females (χ^2^ = 36.38, *p* < 0.001). In addition, though results were not significant for luck, male *observers* attributed ability more to male *actors* than female *actors* [*F*(1,144) = 7.952, *p* < 0.010], and attributed the success of female *actors* to either an ease of task [*t*(1,144) = 3.160, *p* < 0.010] or greater effort [*t*(1,144) = 1.92, *p* < 0.060]. Though ability and luck were not significant, female *observers* attributed an ease of task more to male *actors* than female *actors* [*F*(1,144) = 9.099, *p* < 0.010], and attributed more effort to female *actors* than male *actors* [*F*(1,144) = 7.540, *p* < 0.010]. There were significant interactions between *observer* sex and *actor* sex for ability [*F*(1,144) = 7.953, *p* < 0.010] and task-difficulty [*F*(1,144) = 11.533, *p* < 0.010] attributions, but not for effort and luck.

In a RCT conducted in the laboratory, Heilman and Guzzo (1978) also found no impact of luck as a function of gender. A 2 × 4 factorial design was used to explore whether attribution processes mediate biased reward allocation across the sexes. 15 male and 14 female participants were randomly assigned to a male or female condition. *Actor’s* sex was denoted male or female by sex-casted names. Varying causal explanations were given for the success of four *actors*. Conditions were simulated across explicit dimensions of self-defined characterizations of ability, effort, task-difficulty, and luck, scored on a 9-point scale ranging from ‘*very appropriate*’ to ‘*very inappropriate*.’ Participants were tasked with evaluating the appropriateness of several personnel actions, counting promotions and pay rises, based on a fictitious excerpt with supervisor ratings of the *actors’* performance, ability, and effort. There were no significant main effects for ability, effort, task-difficulty, or luck, nor interactions relating to sex biases. Attributions of success that were biased against females impacted the degree to which rewards were viewed as appropriate and it influenced the magnitude of the reward. But males were disadvantaged in the same way when attributions of success were made toward males in stereotype-contrastive roles. Evidence of sex biases dematerialized when similar causal explanations were given for male and female success. *Post hoc* tests for pairs of factor level means revealed that reward allocation was deemed appropriate when success was predicated on ability and effort, rather than task-difficulty, or luck (*p* < 0.010). Promotion was only considered appropriate when success was ability based (*p* < 0.010), and the preference for reward was greater in the ability over effort condition [χ^2^(1) = 12.76, *p* < 0.001]. Thus, *actors* high in ability, irrespective of sex, were given ratings substantially positive in valence.

Sherrod and Goodman (1978) did not find an impact of luck as a function of gender in a laboratory study. The authors used a one-way ANOVA with a 2 × 2 × 2 design to investigate the role of sex in interpersonal and intrapersonal attributions of success. 24 male and 96 female participants, self-evaluated and evaluated the success or failure of female *actors*. *Actors* were observed attending to a series of 15 spatial reasoning tasks that required matching unfolded geometric outlines to three-dimensional figures. Fictitious performance outcomes were disclosed; the success condition with 12:15 correct answers placed some in the 88th percentile; the failure condition with 5:15 correct answers placed others in the 20th percentile. Attributions were measured on two 15-point scales; relating to how well performance reflects general ability and the extent to which performance was affected by extraneous situational factors (i.e., luck). Actual performance did not differ across conditions [*F*(2,96) = 0.42, *p* < 0.050]. Actors made more ability-based intrapersonal attributions in the presence of male observers than when observed by females [*F*(1,44) = 8.88, *p* < 0.010]. Congruently, an orthogonal contrast determined that male observers made more ability-based attributions than female observers [*F*(1,44) = 4.28, *p* < 0.050]. Thus, both interpersonal and intrapersonal ability attributions were influenced by sex. However, there were no significant effects of gender on luck.

In contrast to the four earlier received studies, Jabes’s (1980) RCT, occupational field-based study, found an impact of luck as a function of gender, but the bias was opposite to that what was expected. In a 2 (*actor_*sex) × 2 (*actor*_age) × 3 (*actor*_occupation) factorial design study, 144 female public and private sector managers (*M*_age_ = 35, range = 22–61) with years of managerial experience between them ranging 1–18 years (*M*_age_ = 4) were recruited. Participants were to explicitly make attributions on self-defined characterizations of ability, effort, task-difficulty, and luck through a four-segment circle divide; each segment therefore varied by the perceived importance of each component. Attributions were predicated on 12 fictitious letters of recommendation and personnel files. The personnel files varied by forename (i.e., David/Joan), age (i.e., 32/46), address, occupation (i.e., Architect/Editor/Social Worker, with equivalent educational details), income range, marital status, and number of children, along with spouse name and occupation. Files were otherwise identical. As compared to males, female success was attributed more to ability [*F*(1,132) = 5.62, *p* < 0.020], and males were thought to be luckier [*F*(1,132) = 3.52, *p* < 0.06].

In a RCT, conducted in the laboratory, Yarkin et al. (1982) found luck attributions were a function of gender, but intersectionality was salient, as attributions of success were evaluated on the intersection of sex and race. (Race differences are later described). 120 participants, male and female, read a description of a highly successful 27-year-old banker, who depending on the condition was male or female, black or white. A cover letter defended the promotion request, highlighting 3 years of banking experience and the *actor’s* occupational history. The *actor’s* sex was denoted by sex-casted names. Explicit attributions were made on self-defined characterizations of ability, effort, task-difficulty, and luck. No significant sex of observer differences were obtained in any of the attribution measures. But main effects for the sex of the *actor* were found for ability [*F*(1,112) = 4.64, *p* < 0.050], effort [*F*(1,112) = 5.76, *p* < 0.050], and luck [*F*(1,112) = 16.67, *p* < 0.001], though not for task-difficulty. In addition, significant interaction effects were seen between *actors’* race and gender on ability [*F*(1,112) = 4.24, *p* < 0.050], effort [*F*(1,112) = 3.96, *p* < 0.050], and luck [*F*(1,112) = 9.35, *p* < 0.010], but not task-difficulty. A Newman–Keuls multiple comparisons test indicated that the success of white male *actors* was attributed more to ability and less to effort or luck, compared with both female *actors*, irrespective of race, and black male *actors* (*p* < 0.050).

Reid et al. (1986) found an impact of luck as a function of gender, by exploring the mediating role of attributions in sex biased interviewing processes in a laboratory-based RCT. 89 male and 91 female participants were randomly assigned to one of six conditions, in which they were to evaluate an interview transcript of male or female *actors* applying for teaching roles in English, History or Mathematics (sex-typed feminine; neutral; masculine, respectively). This was to be assessed against an explicit quadchotomy of self-defined characterizations of ability, effort, task-difficulty, and luck. A 2 (*observer_*sex) × 2 (*actor*_sex) × 3 (*actor*_occupation) three-way ANOVA revealed no main effects or interactions on the overall evaluations. However, a stepwise regression for the overall evaluation of all attributions was significant. Significant three-way interactions were found for effort [*F*(2,168) = 4.64, *p* < 0.010]; task-difficulty [*F*(2,168) = 4.41, *p* < 0.010], and luck [*F*(2,168) = 4.21, *p* < 0.020], but not for ability. In the luck condition, as compared to males (*M* = 2.53; ± 1.41), the past successes of female *actors* (*M* = 3.67; ± 1.50) in the English role received greater attributions to luck. However, in the effort condition, male *observers* attributed more effort to female *actors* (*M* = 4.71; ± 1.64) than male *actors* (*M* = 3.27; ± 1.58) for the Mathematics role. Thus, in the field that was stereotype-contrastive (e.g., a female in a Mathematics role), attributions were biased toward effort, but in a field that supports *a priori* beliefs about traditional gender roles attributions were biased toward luck. These effects were a function of the *observer’s* sex, as there were no significant differences among female *observers*, while male *observers* differentially attributed the success of male and female *actors* as a function of teaching role, on dimensions of effort and luck. However, as it pertains to task-difficulty, both *observers*, male (female *actor* – History: *M* = 3,31; ± 1.25; Mathematics: *M* = 2.21; ± 0.80) and female (male *actor* - History: M = 3.27; ± 1.22; Mathematics : M = 2.71; ± 1.05), agreed that it was easier for *actors* to achieve success in neutral fields than in stereotype-contrastive roles.

Almeida and Kanekar (1989) found an impact of luck as a function of gender in a laboratory-based study. Authors used a 2 (*observer_*sex) × 2 (*actor*_sex) × 2 (*actor*_occupation [bank manager (high-status role)/personal assistant (low-status role)]) × 2 (*actor*_outcome [success/failure]) factorial design, to test whether attributions of success varied according to sex-typed occupations. 336 participants, male and female, were provided with fictitious bibliographical information of *actors*, and were to explicitly attribute *actor* success on a four-point scale that reflected self-defined characterizations of ability, effort, task-difficulty, and luck. The product-moment correlations for pairs of causal elements between ability and luck were 0.14 (*df* = 334, *p* < 0.050). Female *actors* in low-status roles were attributed less to ability by male *observers* than female *observers* (*t* = 2.62, *p* < 0.010). Male *actors* in high-status roles were attributed more to ability than female *actors* in the same role (*t* = 2.38, *p* < 0.020), but male *actors* in low-status roles were attributed less to ability than female *actors* in the same role (*t* = 2.31, *p* < 0.050). Congruently, male (High-status: *M* = 6.10; Low-status: *M* = 5.36; *t* = 3.26, *p* < 0.005) and female (High-status: *M* = 5.38; Low-status: *M* = 6.05; *t* = 3.18, *p* < 0.005) *actors* received higher ability attributions in stereotype-consistent roles. Overall, male success was more highly attributed to effort (*M* = 6.02) than female success (*M* = 5.95; *t* = 2.62, *p* < 0.010) and males in low-status roles (*M* = 5.55), but women in high-status roles (*M* = 5.17) were perceived to have applied less effort than males in low-status roles. Male *actors* in a high-status role (*M* = 3.26) received less attributions to task-difficulty than female *actors* in the same role (*M* = 4.14; *t* = 2.52, *p* < 0.020). But the success of female *actors* was attributed more to task-difficulty when in high-status roles (*M* = 4.14) than when in a low-status role (*M* = 3.36; *t* = 2.25, *p* < 0.050). Critically, females in high-status roles (*M* = 3.41) were considered luckier than males in the same role (*M* = 2.48; *t* = 3.18, *p* < 0.005). But males in low-status roles (*M* = 3.10) were considered luckier than females in the same role (*M* = 2.26) and males in high-status roles (*M* = 2.48; *t* = 2.86, *p* < 0.005). In addition, female *actors* were thought to be luckier in high-status roles (*M* = 3.41) than when in low-status roles (*M* = 2.26; *t* = 3.92, *p* < 0.001). But males in low-status roles (*M* = 3.10) were thought to be luckier than males in high-status roles (*M* = 2.48; *t* = 2.12, *p* < 0.050).

An occupational field-based study, using a matched case control design, conducted by Greenhaus and Parasuraman’s (1993) found an impact of luck as a function of gender only on the intersect of race. The paper was part of a larger study on the career experience of 317 male, 423 female, and eight non-gender stipulated black and white American managers (*M*_age_ = 38.76, ± 8.20) from the fields of banking, communication, and electronics (race differences are later described). A matched sampling process was used to match participants by age, organizational tenure, job function, and seniority, together with their supervisors’ details, to insure against confounding. Performance was rated ‘*satisfactory*’ or ‘*unsatisfactory*’ to invoke the success and failure conditions. Success was explicitly attributed to ability, effort, task-difficulty, support, and luck on a five-point scale. The authors determined that the success of females (*M* = 3.97) was less likely to be attributed to ability than the performance of males (*M* = 3.90; *t* = 2.47, *p* < 0.020). However, this result was true only where it related to highly successful managers. Among moderately successful managers, the performance of females was as likely to be attributed to ability as that of males. No gender differences in attributions were found for effort, task-difficulty, support, or luck, but an interaction between gender and race was observed for task-difficulty (*r* = 0.66, *p* < 0.010) and luck attributions (*r* = 0.39, *p* < 0.050).

Taylor et al. (1993) did not support that gender biases attributions of success toward luck, in their occupational field-based study. Each of the 60 males (aged 15–17) and 60 females (aged 14–50) were provided with two scenarios: a success and failure condition that was factorially manipulated to account for sex and career sex-typing. Explicit attributions were made on self-defined characterizations of ability and luck. Results determined that success was mostly attributed to ability irrespective of the *actor’s* sex or the sex-typing of roles. There were gender differences in ability and luck attributions, but only as it pertained to failure. Significant interactions between the *actor’s* gender, the sex-typed role, and success or failure, revealed that the *actor’s* gender influenced attributions when the failed task was stereotype-consistent [*F*(1,232) = 13.44, *p* < 0.001] and when the succeeded task was stereotype-consistent [*F*(1,232) = 11.37, *p* < 0.001]. No other main effects or interactions reached significance.

In a RCT laboratory-based study, Hill and Augoustinos (1997) were unable to support that luck attributions depended on gender. 26 male and 78 female participants (*M*_age_ = 21.59, ± 5.12) explicitly evaluated attributions on self-defined characterizations of ability, effort, task-difficulty, and luck, through 12 random scenarios. The scenarios indicated male and female success and failure, four attribution questions, and a question to gauge social desirability. A 2 (*observer*_sex) × 2 (*actor*_sex) × 3 (*actor*_course: nursing [feminine]/engineering [masculine]/law [neutral]) × 2 (*actor_*outcome: success/failure) × 4 (*actor*_attribution: ability/effort/task-difficulty/luck]) repeated measures ANOVO revealed a main effect for attribution [*F*(3,312) = 225.16, *p* < 0.001, η^2^ = 0.68] and outcome [*F*(1,104) = 14.43, *p* < 0.001, η^2^ = 0.12]. The interaction between *actor* sex, attribution, and outcome was not significant [*F*(3,312) = 2.47, *p* = 0.062]. Luck was the least made attribution (*M* = 3.90, ± 0.64), followed by task-difficulty (*M* = 3.38, ± 0.58), then ability (*M* = 2.71, ± 0.64), with the most prevalent attributions being made for effort (*M* = 2.01, ± 0.55). The interaction between *observer* and *actor* sex was significant [*F*(1,104) = 6.19, *p* < 0.050, η^2^ = 0.06]; revealing that female *observers* made stronger attributions overall than did male *observers*. Ability attributions were more readily made for success in law (*M* = 2.25, ± 0.82) and engineering (*M* = 2.32, ± 0.83), rather than for success in nursing (*M* = 2.39, ± 0.77). Remarkably, attributions for task-difficulty were made more for success in nursing (*M* = 3.68, ± 0.72) than for engineering (*M* = 3.81, ± 0.76) and law (*M* = 3.83, ± 0.79). Gender associations with attributions of effort and luck were not significant.

Sieverding and Koch (2009) did not find that luck attributions were a function of gender, in a laboratory-based RCT involving computer related tasks. 99 male and 107 female participants (*M*_age_ = 23.7, ± 4.2) were randomly assigned to one of two experimental conditions. They evaluated *actors* who were either male (49:53 male to female *observers*) or female (50:54 male to female *observers*). Participants watched a video of *actors* solving a complex, time-restrictive computer task, and were advised that while 66% were successful, the *actor* they were to evaluate performed well above average. After an explicit attribution of the *actors’* success to either self-defined characterizations of ability or luck, participants had to judge their own hypothetical competence, as compared to *actors*. Irrespective of the *observers’* gender *actors* were expected to be successful [χ^2^(2,206) = 0.18, *p* = 0.910]. There were also no significant gender effects seen when the actors’ sex differed [χ^2^(2,206) = 1.4, *p* = 0.510]. The 2 × 2 multivariate analysis of variance (MANOVA) revealed that attributions of success for female *actors* was not attributed more to luck than male *actors* [*F*(2,202) = 1.24, *p* = 0.270, η^2^ = 0.01], even when accounting for the observers gender [*F*(2,202) = 0.24, *p* = 0.620, η^2^ = 0.00]. Therefore, the posit that female success would be attributed to luck over ability was not supported, nor was there any evidence that the competence of a female would be underestimated.

## Gender discussion

Early reports of gender biases in attribution have ignited efforts to further egalitarian attitudes toward women (Taylor et al., 1993), but this review does not capture or reflect these issues. The gender-levied interpersonal attribution studies within our review spanning 1974–2009 suggest few instances of an invocation of luck for women, and mixed results for ability with effect sizes relatively small in magnitude. There was a center cluster of five associations relating to luck between the 80’s and the early 90’s, although three of those had unexpected findings. First, where luck was more salient to men (Jabes, 1980), and second, where the intersect of gender and race was critical to the outcome (Yarkin et al., 1982; Greenhaus and Parasuraman, 1993). Therefore, only two out of a total of 12 studies support that the success of women is attributed more to luck than men, and of these, one is a RCT and both are laboratory-based studies. This indication of change toward more egalitarian attitudes is promising but could equally reflect social adaptation and a concealment of ideals that are no longer socially acceptable, rather than a change in attitude or reduced biases. In addition, though there was an even divide, we were not able to extrapolate from the survey of evidence a contrast between early and more recent studies as it pertains to ability attributions. Four laboratory-based and two occupational field-based studies found effects on ability. Of these, three were RCT and one was a matched case control study. Overall, results weighed toward no gender effects for luck and ability attributions together, but design differences between studies, especially relating to participant characteristics, methodology, and time, likely account for much of the heterogeneity in results.

A compelling amount of extant literature has converged on the understanding that the success of women is, by and large, undermined by a prevalence of attributions toward luck over ability (e.g., Deaux and Emswiller, 1974; Reid et al., 1986; Heilman and Haynes, 2005), but the present review reveals that context matters. Overall, we find results to be dependent on the study environment and era in which the study was conducted, together with the sample and design. Laboratory experiments, for example, present issues of ecological validity since they have limited generalizability to real-world observations. That said, earlier research from the fields of behavioral economics and psychology have effectively extrapolated from lab-based experimental paradigms (Russell and Fiske, 2008). Intersectionality and the background culture of the sample are equally unobserved confounders that likely underpin differences between studies (Table 2). However, this can be difficult to disentangle. For instance, white women are known to have dissimilar social experiences to black women in different countries, but this can differ by culture, and the demographic status of those making the attribution (Biernat and Kobrynowicz, 1997; Hassan, 2017; Chance, 2022).

Even though associations with luck, specifically, were not significant, Heilman and Guzzo (1978) highlighted the liability of situationally derived causal attributions of success beyond the laboratory environment. This suggests that the preference toward rewarding dispositional factors, without regard to situational factors, has implications on sex differentials in ability attributions. This was evidenced by half of our review studies and has been demonstrated elsewhere (e.g., Yarkin et al., 1982; Sieverding and Koch, 2009). In such instances, women were seen as less able, and were more likely to be overlooked for the most meaningful rewards. By contrast, men were seen as being instrumental in their own success, so attributions leant toward the dispositional, with consequential rewards being more likely. Indeed, although we were unable to evidence gender differences in attributions of success – pointing to a shift towards more egalitarian perceptions – absence of evidence is not evidence of absence, and numerous studies contrast this position (e.g., Sherrod and Goodman, 1978; Greenhaus and Parasuraman, 1993; Biernat and Kobrynowicz, 1997), suggesting again that context matters.

Curiously, two of the five studies with an effect of gender on luck attributions (in either direction) had predominant or exclusive female participation. The remaining three had an equal male to female ratio, as did five of the seven studies that indicated no effect. This could point toward variations in luck attributions being influenced by the gender differences of those observing, rather than of those being observed. However, this should be interpreted with caution as most results were not stratified by participant gender, which would be required to substantiate this observation. It is equally plausible that luck attributions made by women are less prone to socially desirable responses (Hill and Augoustinos, 1997) or, as was shown by Sherrod and Goodman (1978), men generally attribute success more to ability than women do. Equally, male participants may have sought to demonstrate politically correct attitudes and, therefore, refrained from making discriminatory assertions relating to a woman’s performance (Taylor et al., 1993). This would suggest that a latent bias still exists but that it is hard to measure.

Congruent with the dominant view that sex biases ability attributions (e.g., Schein, 1973; Cohen and Bunker, 1975; Taylor and Ilgen, 1981), Sherrod and Goodman (1978) and Greenhaus and Parasuraman (1993) found effects for both luck and ability. However, neither Reid et al. (1986) or Almeida and Kanekar (1989) found main effects nor interactions between gender and ability attributions, despite the significant interactions found for luck. This is of particular interest, as it puts into question the salience of luck across studies and could underlie null findings within the laboratory settings. Ability is more ubiquitously used in discourse than notions of luck, but most studies allowed for luck to be explicitly self-defined, which may not have adequately evoked the condition in the same way that ability was. Indeed, Sherrod and Goodman (1978) submit that men attribute success more to ability than women, while Taylor et al. (1993) found that, irrespective of gender, success was attributed more to ability than other conditions.

In line with Sieverding and Koch (2009), Taylor et al. (1993) submitted that their null findings in attribution differences between genders, suggest a shift in the perception of women, specifically, that the concept of women as skillful professionals may have gained traction as a norm. However, there is nuance to these findings. Reid et al. (1986) submit that non-significant ability results may have been a consequence of setting ability levels so high that ability attributions would be difficult to deny. This supports a view that women are only credited with their success at exceptionally high levels of performance and achievement. An alternative interpretation to this phenomenon is that they must work harder to have their success credited to them over being considered lucky. Biernat and Kobrynowicz’s (1997) findings are an adjunct to this narrative, where lower minimal competency standards but higher ability standards were set for women over men. This can result in an enigma, consistent with the status characteristic perspective, where women experience an ease in meeting low standards and must do more to prove their ability, but have a difficulty in proving their success was a result of ability over luck – a “*twice as hard to be considered half as good*” effect (Biernat and Kobrynowicz, 1997, p. 550). It also contradicts Greenhaus and Parasuraman (1993) and prior studies (e.g., Deaux and Emswiller, 1974; Etaugh and Brown, 1975; Cash et al., 1977) who determined that the success of women was likely attributed to luck when assessing highly successful managers. There, ability was a less salient cue in the consideration of promotion, so women were less likely to be advanced in their career – metaphorically conceived as the ‘glass ceiling.’ Intriguingly, even among moderately successful managers, the success of women was as likely to be attributed to ability as men. These together suggest that gender biases are evoked when a women’s success violates (exceeds specifically) expectations and contradict *a priori* beliefs, where adverse attributions are exploited as a dissonance reduction technique.

Females were evaluated more positively for feminine type-casted roles, with a greater salience for males and masculine type-casted roles. The inference is that women were considered good relative to other women (Jabes, 1980), but not so on a scale with men (Reid et al., 1986). But, while gender was not associated with luck attributions in Feldman-Summers and Kiesler’s (1974) study, it is intriguing that they were unable to determine a single occupation in which women were expected to be more successful than men. A women’s success is, it seems, less anticipated and more likely to be explained through alternative attributional terms, such as luck. In line with predominant sex-casted stereotypes, Almeida and Kanekar (1989) revealed that women were believed to be able in low-status occupations, whereas high-status roles were at the exclusive preserve of men. Jabes’s (1980) findings might seem evidence to the contrary, but this study cued more to individuals being in management, rather than the peculiarities or the prestige of the occupation.

## Race

We survey seven studies in total, five of which exclusively investigated race as a key influencer of interpersonal attributions of success, with an additional two studying the intersection of sex. Of those, five were RCT and one was a matched case control study. Five studies were conducted within a laboratory setting and two were conducted in an occupational field-based setting. We follow this up with a summary of evidence, ordered by publication date, as a way to understand the psychological mechanisms that underlie attribution errors in success as they pertain to race. A result summary can be found in Table 1.

The first study to consider race and luck attribution is owed to Greenberg and Rosenfield (1979). Overall, these authors found that luck attributions were a function of race in a RCT laboratory-based study. They examined the moderating role of ethnocentrism^1^ over stereotype utility in racially driven interpersonal attributions of success. Ethnocentrism and discrimination were highly correlated (*r* = 0.54, *p* < 0.010). 116 white male participants were shown an audiovisual of two *actors* performing well and two performing poorly on a test. The test consisted of ten trials where the *actor* guessed the shape or line count on hidden cards, followed by a questionnaire with scales on dimensions of ability, effort, task-difficulty, and luck that were explicit and self-defined. One MANOVA revealed a significant main effect for *actor* outcome on all four attribution dimensions (*p* < 0.001), but success was attributed less than failure. A second MANOVA revealed a significant main effect [*F*(1,45) = 5.63, *p* < 0.050] where participants high in ethnocentricity attributed the success of black *actors* less to ability (*M* = 1.78) and more to luck (*M* = 1.22) than the success of white *actors* (Ability: *M* = 2.35; Luck: *M* = 0.22). Participants low in ethnocentricity attributed the success of black *actors* more to ability (*M* = 2.46) and less to luck (*M* = 0.29) than the success of white *actors* (Ability: *M* = 2.13; Luck: *M* = 0.63). The interaction between ethnocentrism, *actor* outcome and *actor* race was significant on attributions of ability [*F*(1,46) = 8.67, *p* < 0.005], but not so for attributions of luck [*F*(1,46) = 3.11, *p* < 0.090].

Orpen (1981) found that luck attributions were a function of race within a RCT, occupational field-based study. The author evaluated whether attributions of success, made by 136 white middle-managers (*M*_age_ = 32.6) on four black and 26 white subordinates was influenced by race. The racial divide of the *actors* corresponded to the racial balance in South Africa. Managers were asked to evaluate performance information indicated on six indices (i.e., output quality and quantity; turnover and absenteeism; subordinate grievances; and financial/budget control) for *actors* over the last year. Attributions were explicitly made on self-defined characterizations of ability, effort, task-difficulty, and luck. Actual performance varied widely, except in the case of four *actors*; two black and white, who had identically good performance, and two other black and white *actors* with identically poor performance. A comparison was made by *t*-tests for independent samples between the *observers’* mean ratings of the causal factors in the success condition for black and white *actors*. Ability (*M* = 4.86) and effort (*M* = 5.02) were given less credence for the success of black *actors* than their white counterparts (Ability: *M* = 6.10; Effort: *M* = 6.04, *p* < 0.01). While task-difficulty (*M* = 8:10) and luck (*M* = 8.08) were deemed more critical to the success of black *actors* than white *actors* (Task-difficulty: *M* = 4.87; Luck: *M* = 5.51, *p* < 0.001). Failure followed a divergent pattern of attributions, with dispositional characteristics being most salient among black *actors*.

In a laboratory-based RCT, Whitehead et al. (1982) found that luck attributions were a function of race. 77 black and 43 white participants were randomly provided with a brief description of 156 black and 208 white *actors* in college who were to be rated on a series of characteristics, including intelligence and athleticism. Attributions were made explicitly on self-defined characterizations of ability, effort, task-difficulty, and luck. A multivariate analysis revealed a significant interaction between success/failure, *observer* race and *actor* race [*F*(4,170) = 2.39, *p* < 0.052]. In the academic condition, univariate tests (*df* = 1,173) showed that *observers* attributed success more to ability (*M* = 5.17, *F* = 56.29, *p* < 0.001) and effort (*M* = 5.63, *F* = 50.32, *p* < 0.001) than task-difficulty (*M* = 2.91, *F* = 5.39, *p* < 0.030), but not to luck (*M* = 2.35, *F* < 1). In the athletic condition, *observers* attributed success more to ability (*M* = 5.51, *F* = 56.68, *p* < 0.001) and effort (*M* = 5.46, *F* = 48.32, *p* < 0.001), than task-difficulty (*M* = 3.13, *F* = 7.95, *p* < 0.005) or luck (*M* = 2.86, *F* = 4.78, *p* < 0.030). Though not significant in the former, a 2 × 2 ANOVA indicated that black *actors* were thought to be less intelligent [Black: *M* = 59.84; White: *M* = 66.95, *F*(1,114) = 2.92, *p* < 0.090], but more athletic [Black: *M* = 70.33; White: *M* = 57.37, *F*(1,114) = 12.15, *p* < 0.001] than their white counterparts. Also borne out of the data, black *observers* more generously attributed academic success to ability when the *actor* was part of ingroup [Black: *M* = 5.84; White: *M* = 4.93, *F*(l,173) = 2.98, *p* < 0.086]. This result was not replicated when *observers* were white who, regardless of race, attributed to academic ability equally (Black: *M* = 5.11; White: *M* = 4.94, *F* < 1). Attribution differences in the athleticism success condition were not seen irrespective of whether the *observer* or *actor* was black or white [Black/White: *M* = 5.96; Black/Black: *M* = 5.85; White/Black: *M* = 5.11; White/White: *M* = 5.19, *F*(l,173) = 2.98, *p* < 0.086].

Yarkin et al. (1982) could only support that luck attributions for success were a function of race on the intersect of gender, in their RCT laboratory-based study. 120 male and female American participants were randomly assigned to each condition using a 2 (*actor*_gender) × 2 (*actor*_race) × 2 (*observer_*gender) between-subjects factorial design. Attributions were made explicitly on self-defined characterizations of ability, effort, task-difficulty, and luck. Race was denoted by résumé information, including affiliations (e.g., associations to historically black or white American universities). Main effects for the race of the *actors* were seen for ability [*F*(1,112) = 10.13, *p* < 0.001] and effort [*F*(1,112) = 5.12, *p* < 0.05], but not so for task-difficulty or luck. *Observers* attributed success significantly more to ability and effort when the *actor* was a white male, relative to being a white female, or a black person irrespective of gender (*p* < 0.05 in all cases). Interactions between the *actors’* race and gender were significant for ability [*F*(1,112) = 4.24, *p* < 0.050], effort [*F*(1,112) = 3.96, *p* < 0.050], and luck [*F*(1,112) = 9.35, *p* < 0.010], but not for task-difficulty.

In an occupational field-based, matched case control study, Greenhaus and Parasuraman (1993) found an impact of luck attributions as a function of race. 322 black and 426 white American managers (*M*_age_ = 38.76, ± 8.20) in banking, communication, and electronics participated. There was a higher proportion of women among black participants compared to white participants (gender differences earlier described). Performance was rated ‘*satisfactory*’ or ‘*unsatisfactory*’ to invoke the success and failure conditions. Success was attributed to ability, diligence, luck, task-difficulty, and support on a five-point scale. The authors determined that the promotional prospects of black managers were less promising than their white counterparts (*r* = 0.06, *p* < 0.050). Overall, ability attributions were weaker for black (*M* = 4.10) than white managers (*M* = 4.24). A similar pattern was observed between black (*M* = 3.96) and white (*M* = 4.14; *t* = 2.14, *p* < 0.050) managers with limited experience (≤1 year). But among experienced managers (>1 year), ability attributions did not significantly differ. Effort attributions were weaker for black (*M* = 4.13) than white (*M* = 4.27) managers, but no differences were found regarding managerial experience. Congruently, black managers (*M* = 2.35) were thought to have received more support than white managers (*M* = 2.17). Significant gender and race interactions were found for attributions to task-difficulty (*p* < 0.010). Overall, black male managers (*M* = 1.70) were thought to have easier work than white male mangers (*M* = 1.33). But white female managers (*M* = 1.45) were thought to have easier work than black female managers (*M* = 1.32, *p* < 0.050). Among managers with limited experience, black male managers (*M* = 1.64) were also thought to have easier work than white male mangers (*M* = 1.40, *t* = 2.86, *p* < 0.010). But task-difficulty attributions did not differ among experienced managers. Significant gender and race interactions were found for luck attributions (*p* < 0.050). Overall, the success of black male (*M* = 1.40) and black female managers (*M* = 1.27) was more likely to be attributed to luck than their white male counterparts (*M* = 1.23; *p* < 0.050), but no differences were found by managerial experience. Negative attributions were reduced when the supervisor-supervisee relationship was strong and longer than a year.

Knight et al. (2003) found that race influenced luck attributions in their laboratory-based study of aversive racism^2^, by way of stereotype-consistent and stereotype-contrastive patterns in attributions. 156 white males participated in this 2 (*actor*_race: black/white) × 2 (*actor*_status: subordinate/manager) × 2 (*actor*_mistake: small/large) between-subjects study. Status represented dimensions of success or failure. Participants were to act as middle-level managers who reported to higher-level managers and managed subordinates. *Actors* varied only by status and the magnitude of the mistake made. Luck was leveraged on stereotype-consistent or contrastive factors beyond the *actors’* control. Ambivalent information was included to increase the possibility that participants would pursue schema-consistent evidence in their decision process. Evaluations were made on several dimensions, including work quality, stress tolerance, leadership, potential, interpersonal skills, conceptual skills, administrative skills, and promotion. Using a between-subjects ANOVA with a sequential Bonferroni correction to control the Type-I error rate, a two-way interaction was found for effort [*F*(1,145) = 7.62, *p* = 0.007, η^2^ = 0.050]. A three-way interaction between race, status, and mistake made was not significant, but the multivariate analysis of variance (MANOVA) revealed a two-way interaction between race and status [*F*(9,137) = 2.20, *p* = 0.026, η^2^ = 0.130]. Specifically, negative attributions of black leaders and white subordinates were stereotype-contrastive, but positive attributions for black subordinates and white leaders were stereotype-consistent. Stereotype-contrastive patterns in attributions were attributed to luck. Congruently, white managers (*M* = 3.67, ± 1.06) received stronger ability attributions than white subordinates [*M* = 3.20, ± 1.08; *t*(74) = −1.92, *p* = 0.029]. But this normative pattern was reversed for black managers (*M* = 3.37, ± 0.85) and subordinates [*M* = 3.86, ± 1.07; *t*(76) = 2.23, *p* = 0.015].

In a laboratory-based RCT, Ellis et al. (2006) determined that luck attributions by subordinates were not impacted by leader race in the expected direction; a reversal of anticipated effects was seen, with attributions toward black leaders being more ability-based than white leaders. 123 male and 54 female participants were randomly assigned to four-person groups, with one leader and three subordinates. 162 participants were white and 15 did not disclose race. In an airspace simulated decision-making task, subordinates independently collated information to determine the level of threat that an aircraft represented. Said information was relayed to the leader who made an overall decision that was judged by the subordinates. Group members were informed of the quality of that decision. On the basis of their performance, the top performing team members could earn a bonus. Attributions were explicitly made on characterizations of intelligence denoting ability, along with effort, task-difficulty, and luck on a five-point scale. Consistent with the fundamental attribution error, as leader performance increased, observers were more likely to attribute outcomes internally (e.g., ability; *r* = 0.47, *p* < 0.010) and less likely to attribute toward external causes (e.g., luck; *r* = −0.59, *p* < 0.01). In the case of high-performance teams, a three-step hierarchical regression revealed that black leader performance was attributed to ability (*M* = 2.35, ± 2.34), more than white leaders [*M* = 0.72, ± 1.98; *t*(88) = 3.56, *p* < 0.010]. But no significant differences in ability attributions were found between the races when team performance was low. Although the interaction between leader race and performance explained a significant proportion of the variance in ability attributions (Δ*R*^2^ = 0.02, *p* < 0.010), in the case of low-performance teams, black leader performance was attributed to luck (*M* = 2.08, ± 2.20) more than for white leaders [*M* = 1.25, ± 2.34; *t*(85) = 1.73, *p* < 0.05]. But in high-performance teams, the success of black leaders was less attributed to luck (*M* = −2.03, ± 2.33), than white leaders [*M* = −1.18, ± 2.03; *t*(88) = −1.85, *p* < 0.050]. Irrespective of race, high-performing managers received higher ratings when their performance was attributed to ability over luck, but low-performing managers received higher ratings when their performance was attributed to luck.

## Race discussion

All seven race-related studies within this review, from 1979 to 2006, provided evidence of attribution errors, but two studies had unexpected findings. As it pertains to luck and ability attributions, with one exception (Ellis et al., 2006), attribution errors were in the expected direction. In addition, one study only demonstrated attribution errors with respect to luck on the intersect of race and gender (Yarkin et al.’s 1982). Thus, five out of a total of seven papers suggest that the success of black individuals is attributed more to luck and six suggest their success is attributed less to ability than their white counterparts. While results are weighted heavily in favor of attribution errors being impacted by race – an immutable factor beyond one’s control – and most were in the expected direction, one late study found that attributions were favorable toward black individuals over their white counterparts (Ellis et al., 2006). The only RCT and matched case control studies that were occupationally field-based were Orpen’s (1981) study of white managers, and Greenhaus and Parasuraman’s (1993) study of black and white managers; both that revealed attribution errors as it relates to luck and ability. Though several other laboratory-based studies followed the same trajectory (Greenberg and Rosenfield, 1979; Whitehead et al., 1982; Knight et al., 2003).

There are important implications of Greenberg and Rosenfield (1979), Orpen (1981), Whitehead et al.’s (1982), Greenhaus and Parasuraman (1993), and Knight et al. (2003) findings for organizations. They converge on the understanding that attributions of success are negatively influenced by race and yet, within this context, they each highlight distinctions in the obstacles faced between the races, including whether black individuals are seen as being worthy drivers of their own success, particularly as seniority increases. These offer a partial explanation for an inequity in reward allocation within many organizations (Amis et al., 2018), since rewards are afforded on the basis of individuals being credited for their own success (Wittig et al., 1981). These biases appear to persist across a black person’s career, existing on an increasing level of liability with progression into more senior roles despite evidence of strong ability (Orpen, 1981). However, Ellis et al. (2006) found a reversal of effects, since black individuals were thought to have higher ability and were rated lower in luck than their white counterparts. This purported shift toward more egalitarian values runs counter to expectations and suggests that context matters. The authors argue *post hoc* that, despite this sociopolitical transformation, there remains an inequity in society that develops into “*White guilt*,” and it is this that positively influences attributions toward black individuals in an attempt at restitution (Ellis et al., 2006, p. 312). Also of note, it is possible that untested intersectionality may have confounded the results, because while gender did not affect accuracy-linear consistency or variability bias, it did significantly impact the decision-making error-mean bias. Still, having controlled for gender, the findings remained the same. Interestingly, despite the demonstration of effect modification by gender, Yarkin et al. (1982) found that race influenced ability attributions, but not luck attributions, which suggests that luck indices, that were explicit and self-defined, were likely not as salient as ability.

Different to Greenberg and Rosenfield (1979), Orpen (1981); Yarkin et al. (1982), Knight et al. (2003), and Ellis et al. (2006) had an exclusively white participant pool that may have influenced luck and ability attributions. It is conceivable that reduced social desirability was observed in participant responses in these latter studies, because of a diminished pressure to conform to politically correct attitudes and ideals among one’s ingroup. Ellis et al. (2006) suggest that an awareness of racial biases has important implications for attributions, in that it can create a level of discomfort when making attributions that would privilege certain groups. While not wanting to over-simulate the psychological processes at play, these authors also muse over the potential for social desirability as a way to explain their null results. However, Whitehead et al. (1982) and Greenhaus and Parasuraman (1993) submit that black individuals are not necessarily internalizing these oversaturated messages of racial biases, such that they are implicitly informing their attributions. This was seen in an attributions study that evaluated the level of success, rather than ability versus luck (Hassan, 2017), where race was not a determinant in the interpersonal attributions of success among a pool of exclusively black participants. This suggests that it is not only the race of the *actor* that shapes attributions, but also the race of the *observer*, which is an important consideration for focused interventions. Curiously, there was no available evidence on the interpersonal attributions of exclusively Asian or other ethnic individuals on black individuals, nor a direct contrast between these groups, nor a comparison of these groups against white individuals. In spite of one gender-related study having a South Asian sample, this may be subject to the majority of studies being conducted in America (Table 2), where race is primarily dichotomized between black and white because of the population sociodemographic distribution. These documented differences in attributions between participants who are all white and those who are all black suggest that results cannot be legitimately generalized to other racial groups without further evidence.

Greenberg and Rosenfield (1979), Orpen (1981), Whitehead et al. (1982); Greenhaus and Parasuraman (1993), and Knight et al. (2003) demonstrated that the attributions of success of both black and white individuals are subject to positive in-group bias. Positive ability attributions toward ingroup members could be suggestive of a self-serving bias; used to protect one’s self-esteem by evoking empathy toward the group that one belongs. This is theorized by Kelley (1967) and Regan and Totten (1975) who shared the view that an *observer’s* emotional disposition toward an *actor* is essential to the orientation of the attribution. It also distally aligns with Charles and Littig (1982) and Jones and Nisbett (1987) who stated that the intrapersonal attributions of individuals are skewed toward the dispositional, which is relevant if *observers* are aligning themselves with the *actor*. Linville and Jones’s (1980) polarized appraisal hypothesis purports that information on success has greater impact on outgroup than ingroup evaluations. This would mean that the complex schema that individuals hold on ingroups is weighed by positive and negative features of the ingroup, leading to more moderate evaluations and a realization that the outcome could be attributed to multiple causes.

Throughout this review we see stereotype-consistent and stereotype-contractive patterns of influence. Stereotype utility was greater in Whitehead et al.’s (1982) athleticism condition, where black participants attributed the performance of white *actors* more than that of black *actors* to the relative ease of the athletic task. The success of a white *actor* in the athletic condition created a juxtaposition between the result and the specious stereotype that white people have less athletic ability than black people, whereas failure in this remit would be congruent with said stereotype. Still, irrespective of race, the evocation of luck indices was more prevalent in the athleticism condition than the academic, suggesting that attributions are also task-relative. A similar stereotype consistency paradigm was also observed in Knight et al.’s (2003) study, where black leaders and white subordinates received aversive attributions when compared to white leaders and black subordinates. In both scenarios, stereotype-consistent contexts do not violate *a priori* beliefs as stereotype-contrastive contexts would. Both are in line with the social dominance orientation paradigm, as *observers* appeared to have a partiality toward preserving an imbalanced societal power distribution, rewarding white leaders for academic success and black subordinates for athletic success, yet penalizing the inverse. As a plausible alternative to the attribution theory, Ellis et al. (2006) proposed the expectancy-violation theory (Jussim, 1986) as a justification of this phenomenon, where stereotype-contrastive behaviors are evaluated more extremely in the direction of the violation. However, it has not yet been possible to support this theory empirically. In addition, successful managers have been found to receive higher evaluations, irrespective of race, when their performance was attributed to ability over luck (Ellis et al., 2006), but ineffective managers received higher ratings when their performance was attributed to luck. This stands in contrast to the dominant position in the present review, but is congruent with some earlier literature (Whitehead et al., 1982; Knight et al., 2003).

Ellis et al. (2006) uniquely explicated on the basis of Weiner’s (1980) causal attribution model with a focus on the *locus of control*. It was determined that two discrete attributional processes were occurring; the first, where success is perceived over failure; the second, where race was perceived. The influence of the former process was said to prevail over the latter process, to the extent that the influence of race was moderated and ran counter to view that negative race stereotypes drive negative attributions toward luck and for ability. One could submit that this illustrates a positive shift in the prejudicial attitudes of society and the possible attempts at restitution, or more cynically, a realization that occupational diversification is no longer optional. But Orpen’s (1981) and Greenhaus and Parasuraman (1993) found that the effects of race on attribution career advancement prospects were often implicit and surreptitious, materializing by way of biased attributions of ability and performance evaluations – again, a ‘*glass ceiling*’ effect – such that black managers, male and female, were penalized twice. Prejudice in both the attributions of success (i.e., assessments of low ability and greater luck) and the evaluations of performance (i.e., harsher appraisals) meant black managers were seen as less promotable than their white counterparts.

Racial heterogeneity is expected to increase as more organizations come to realize the moral responsibility and competitive advantages of doing so (Jackson and Joshi, 2001). The shift toward equality will invariably lead to more ethnic individuals securing positions over white individuals, such that evaluations of success may become increasingly more vulnerable to implicit biases (Ellis et al., 2006). However, Greenhaus and Parasuraman (1993) found that negative attributions were less prevalent when the supervisor-supervisee relationship was stronger and longer-term. Thus, relationship and familiarity may prove to be an effective prescription to attenuate biases in interpersonal attributions of success. This temporal effect was said to be a result of the supervisor having sufficient information to come to an authentically impartial judgment on ability. They were also able to observe consistency in performance, such that it mediates the association. However, an alternative explanation is that this is merely a selection effect because greater lengths of employment suggest that the employee is liked.

## General discussion

In a systematic review of literature, we summarize the evidence on whether attributions of success to luck or ability are influenced by sociodemographic differences, viz. gender and race. On the whole, studies within this review have shown some support for Weiner’s (1980) Causal Attribution Model (Table 3). But ultimately context matters. Results largely depended on study design and locale, the characteristics of the sample, and the era in which the study was undertaken. Across both sociodemographic characteristics, there was a stronger, more consistent demonstration of attribution errors related to ability, than to luck, which could plausibly be because indices of luck are less salient to individuals than ability. In the luck condition, attributions were not overall adversely made for women relative to men, and there was an equivalence of evidence in the ability condition, which alludes to an indication of change toward more egalitarian values as it pertains to gender. However, attributions of success were adversely made toward black individuals relative to white individuals in both luck and ability conditions, such that it was harder for them to document their success in the dispositional domain.

**TABLE 3:** Quantitative summary of attribution errors.

	Luck		Ability		Luck/Ability ratio
	Effect observed (✓)	Effect not observed (×)	Effect observed (✓)	Effect not observed (×)	
Gender	3	9	6	6	9:15
Race	5	2	5	2	10:4
Overall	7	12	11	8	**18:20**

Bold value represents the total ratio between luck and ability.

This latter finding is consistent with Berger et al.’s (1966) Expectation States Theory, insofar as interpersonal attributions of success being predicated on little more than spurious and immutable characteristics of an individual. The difficulty in the mapping of performance can lead to systemic reward inequities, and thus, poorer, protracted career outcomes for black individuals. This is echoed by Greenhaus and Parasuraman’s (1993) study, and later evidence (Biernat and Kobrynowicz, 1997), which revealed a juxtaposition between lower minimum standards, but higher ability standards being set for black over white individuals, although, they also found the same for women over men, which sits in contrast to our systematic results.

Essentially, high nominal performance was taken as evidence of high ability, without adjusting for privilege or the ease through which it was achieved, which epitomizes the correspondence bias (or fundamental attribution error^3^). As earlier documented (Ross et al., 1977), social perceptions of participants may have been similarly distorted, such that people failed to adjust for inter-individual advantage and overestimated ability. People can be disillusioned when their expectations are not accurate (Russell and Fiske, 2008). This suggests that implicit processes are underlying these attribution biases. It reflects a genuine belief in the validity of the attribution made, and thus we see an unconscious bias. It is plausible that owing to the fundamental attribution error^1^, observers were ignorant to the extent that race influenced their perceptions, and thus, attributions. This failure of insight has critical implications for person perception accuracy, where perceptions are wrongly justified and falsely credited to other causes.

On the intersect of race and gender, Yarkin et al. (1982) and Greenhaus and Parasuraman (1993) supported that attributions of success were favorable toward white men, as compared to black men, black women, and also white women. Therefore, the direction of the attribution was influenced by intersectionality, but not evenly. Biases that exist on the intersect of gender and race such as these, that limit vertical mobility within organizations are known as the “*concrete ceiling*”; a metaphor that describes the unique obstacles that black and other ethnic women face in advancing into positions of leadership (Chance, 2022). Even so, in the current paper, race differences were more influential than gender differences; though white managers were predominantly favored over black managers, in some cases, women were favored over men.

The correspondence bias can apply to individuals at all levels, as well as groups and organizations. Swift et al., (2013) see this as comparative to politicians who benefit from fortuitous and/or exogenous conditions for which they are not responsible (e.g., initiatives established by a former administration), instead of being rewarded or penalized for action taken by their own constituencies. The failure to discount luck, the exaggeration of ability, and the anticipation of more cross-situational consistency than is reasonable to expect, can be seen as an instance of the correspondence bias, resulting in the fundamental attribution error^1^ (Ross et al., 1977).

There are several adverse individual-level trends within organizational settings that can arise when decision-makers fall victim to the correspondence bias; from lost opportunity to exclusionary behaviors. However, the correspondence bias is subject to a number of moderating factors that could be leveraged to effect change. The emotional disposition between individuals is said to be essential to the orientation of the attribution (Kelley, 1967; Regan and Totten, 1975). Therefore, evoking empathy between groups by promoting alliances and relationships could be an effective way to reduce attribution errors (Silvester and Chapman, 1997). It is also important to consider who to promote to decision-making positions and how to effectively manage them. For instance, more reflective individuals are less likely to make attribution errors^1^ (D’Agostino and Fincher-Kiefer, 1992). This is also true when individuals are made to feel accountable for their behaviors (Tetlock, 1985). Although feelings of elation have been found to have similar effects (Forgas, 1998), feelings of stress, exhaustion or inundation can lead to errors in attributions. This speaks to issues on workload, time autonomy, flexible working practices, and tasks that individuals are not well equipped to handle (Gilbert et al., 1988). Finally, on a macro level, collectivistic societies are less vulnerable to attribution errors of this kind, which presents an persuasive model for organizational culture change that would be less prone to these errors (Morris and Peng, 1994; Silvester et al., 1999; Miyamoto and Kitayama, 2002).

Findings in the present review support Kelley’s (1967) assertion – that success being attributed to ability and failure being attributed to luck – is an over-simplification of the issues at play. Both gender and race have been shown to mediate the direction and strength of attributions made differentially (e.g., Sherrod and Goodman, 1978; Greenhaus and Parasuraman, 1993; Knight et al., 2003). Understanding the psychological mechanisms that underlie attribution errors is crucial to ensuring remedial effects are maximally effective. It is, nonetheless, accepted that the attribution process is highly complex and differs as a function of the relationship between *observers* and *actors* (Orpen, 1981; Charles and Littig, 1982), which is further substantiated by this review.

Ultimately, observed inconsistencies between studies may be a result of the heterogeneity in study design, sample characteristics, context, and the era in which the research was performed. Such inconsistencies create challenge in making parallels and distinctions but, overall, there was stronger evidence of interpersonal attributions of success to luck and ability being predicated these largely static sociodemographic factors. Moreover, despite an indication of change between the genders, contradictory evidence, latent variables that are sporadically addressed, and the challenge of disentangling how intersectionality develops heterogeneous modes of privilege and discrimination, all add to the complexities of bias in attributions of success. This perhaps explains the paucity of interventions offered and the absence of a panacea, which together raise concern about the permanence of these biases.

Results should be considered with respect to some limitations. There were circa 25% more gender-focused studies in this review than race, spanning across eight more years. In addition, most authors recruited participants from America (75%), and almost all papers were published in journals of a psychological or social science nature (Table 2). This skew in distribution among the review papers may have created a bias that contributed, in part, to the divergence in results. Equally, the participant pools within and between studies were limited (Table 1). All studies were restricted to a single or binary gender. It would have been interesting to see whether the race effects found could be extrapolated to other ethnic groups, but no studies used ethnicities beyond black and white. Orpen’s (1981) results, for example, are of particular interest but the exclusively white sample precluded the possibility of exploring race as a moderator or adjustment for in-group favoritism. Further, two review studies (Orpen, 1981; Greenhaus and Parasuraman, 1993) showed that attributional errors in luck and ability remained robust in older ages, which was also later documented (Swift et al., 2013). But the majority of the studies within this review (12 in total) recruited university aged students who may have more egalitarian values than older groups (Sieverding and Koch, 2009). Finally, the literature discussed in this review were all cross-sectional. Longitudinal study would be advantageous for two reasons. First, Greenhaus and Parasuraman (1993) showed that attributions change over time; insofar as the length of the supervisor-supervisee relationship being of importance to the direction and magnitude of the attribution. Therefore, prospective data could be used to test interventions as a way to attenuate the strength and impact of attributions. Second, it may be possible to identify attributional patterns at different stages of the interactions between decision-makers and observed individuals.

## Conclusion

Decades of research in psychological and social sciences have pointed to individuals making systematic errors in attributions of success. This review only partially substantiates this purported consensus, although it adds to the evidence-base that context is critical to whether individuals systematically mistake immutable sociodemographic factors for ability, ignore the role of luck, and ignore the effect of *regression to the mean* (Denrell et al., 2019). What we do see consistently is that these biases underlying attribution errors lead to disparities between opportunities afforded or denied to individuals, and so they have important implications for organization diversification and occupational engagement. Although this review only provides narrow support for the view that those believed to be the most talented in society may merely be the luckiest (Pluchino et al., 2018).

## Data availability statement

The original contributions presented in this study are included in the article/supplementary material, further inquiries can be directed to the corresponding author.

## Author contributions

GL: conception. OH: planning, systematic literature review, literature interpretation, and manuscript draft. Both authors reviewed and contributed to the final manuscript.

## Footnotes

1 Ethnocentrism – using cultural preconceptions originating in the standards and customs of one’s own culture, as a frame of reference to judge other cultures, practices, behaviors, beliefs, and people, instead of using the standards of that particular culture. Ethnocentrism may result in a dislike for the other cultures assessed from that viewpoint.

2 Aversive racism is a subtle, yet insidious form of prejudice that emerges when discriminatory views can be justified by factors other than race where situational norms are ambiguous (Gaertner and Dovidio, 2000).

3 The correspondence bias occurs when individuals draw correspondent dispositional inferences from observed behavior, despite this behavior being highly constrained by situational factors. Even though this phenomenon has been proposed to be multiply determined, the most common explanation is that individuals ubiquitously but erroneously believe that situational factors have little impact on behavior, which is a fundamental attribution error (Gawronski, 2004). In the current content, the correspondence bias, a fundamental attribution error (Ross et al., 1977), theorizes that individuals profligately attribute behavioral outcomes, such as success, to dispositions, such as ability, while ignoring situational mediators, such as luck. Therefore, successes are thought to be an exclusive byproduct of innate abilities, while ignoring the salience of diffuse characteristics, social situs, cultural legacies, and institutional constraints that confound assessments, such as that evidenced by Mitchell and Wood (1980); Mitchell and Kalb (1981), and Gawronski (2004).
